# UVB-Induced Microvesicle Particle Release and Its Effects on the Cutaneous Microenvironment

**DOI:** 10.3389/fimmu.2022.880850

**Published:** 2022-05-06

**Authors:** Timothy C. Frommeyer, Michael M. Gilbert, Garrett V. Brittain, Tongfan Wu, Trang Q. Nguyen, Craig A. Rohan, Jeffrey B. Travers

**Affiliations:** ^1^ Department of Pharmacology and Toxicology, Boonshoft School of Medicine at Wright State University, Dayton, OH, United States; ^2^ Department of Dermatology, Boonshoft School of Medicine at Wright State University, Dayton, OH, United States; ^3^ Department of Medicine, Dayton Veterans Administration Medical Center, Dayton, OH, United States

**Keywords:** microvesicle particles, platelet-activating factor, ultraviolet light, UVB, platelet-activating factor receptor, immunosuppression, local inflammation, aSMase inhibitors

## Abstract

Ultraviolet B radiation (UVB) has profound effects on human skin that results in a broad spectrum of immunological local and systemic responses and is the major cause of skin carcinogenesis. One important area of study in photobiology is how UVB is translated into effector signals. As the skin is exposed to UVB light, subcellular microvesicle particles (MVP), a subtype of bioactive extracellular vesicles, are released causing a variety of local and systemic immunological effects. In this review, we highlight keratinocyte MVP release in keratinocytes in response to UVB. Specifically, Platelet-activating factor receptor agonists generated by UVB result in MVP released from keratinocytes. The downstream effects of MVP release include the ability of these subcellular particles to transport agents including the glycerophosphocholine-derived lipid mediator Platelet-activating factor (PAF). Moreover, even though UVB is only absorbed in the epidermis, it appears that PAF release from MVPs also mediates systemic immunosuppression and enhances tumor growth and metastasis. Tumor cells expressing PAF receptors can use this mechanism to evade chemotherapy responses, leading to treatment resistance for advanced cancers such as melanoma. Furthermore, novel pharmacological agents provide greater insight into the UVB-induced immune response pathway and a potential target for pharmacological intervention. This review outlines the need to more clearly elucidate the mechanism linking UVB-irradiation with the cutaneous immune response and its pathological manifestations. An improved understanding of this process can result in new insights and treatment strategies for UVB-related disorders from carcinogenesis to photosensitivity.

## Introduction

Ultraviolet B (UVB; 290-320nm) radiation found in sunlight is essential for the production of vitamin D in humans (1). However, prolonged exposure can lead to a myriad of pathologic effects including erythema, photoaging, inflammatory responses, and skin cancer (2–4). Within the epidermis, UVB is able to damage DNA in various cell types in the skin, especially the keratinocyte (5–7). The UVB rays induce formation of cyclobutane pyrimidine dimers which have the potential to be propagated to subsequent cellular populations, which may result in pro-carcinogenic changes (8, 9). UVB also has the ability to act as a pro-oxidative stressor, generating various immunoregulatory mediators including prostaglandin E_2_, serotonin, histamines, interleukin (IL)-6, IL-10, tumor necrosis factor-alpha (TNFα) and the lipid mediator Platelet-activating factor (PAF) (10–12). UVB-induced production of these bioactive molecules results in acute inflammation, erythema, cancer, and degenerative aging (13, 14). Moreover, at high doses, UVB-irradiation and subsequent release of PAF and other agents such as TNFα can lead to systemic effects including fever, malaise, and immunosuppression (15, 16). PAF exerts a variety of effects through the G-protein coupled transmembrane Platelet-activating factor receptor (PAFR), which is expressed on multiple cell types including keratinocytes (17, 18). Cutaneous UVB exposure and activation of keratinocytic PAFR results in similar signaling pathways, which suggests that many UVB-induced effects are mediated *via* PAFR or modified by associated PAFR activation (19, 20). Since UVB absorption is limited to the epidermis, bioactive agents that leave the skin are thought to act as effectors for UVB responses.

Platelet-activating factor (PAF) is the term denoting a family of glycerophosphocholine (GPC)-derived lipid mediators implicated in a number of pathologic processes including skin carcinogenesis, liver disease, and allergic rhinitis (16, 21, 22). Although PAF application results in pro-inflammatory processes similar to the acute effects of UVB radiation, it can also produce immunosuppressive effects *via* upregulation of regulatory T cells (10, 16, 17, 23). The synthesis of PAF is associated with several different pathways including the remodeling and *de novo* pathways (24, 25). Of particular interest is the formation of PAFR agonists in response to increased reactive oxygen species (ROS) resulting in non-enzymatic formation of oxidized GPC (ox-GPC) with PAFR agonistic activities (26–28). This has prompted the notion that UVB-irradiation results in activation of the PAF system. The link between UVB, PAFR signaling, and photobiology is further implicated by PAFR activation in the early acute response of UVB as well as UVB-mediated systemic immunosuppression (10, 16, 17, 23, 29, 30). Thus, the PAF system plays an integral role in dermal pathophysiology and skin response to environmental stressors (31). After generation, PAF resides in cellular membranes where it can act upon itself or neighboring cells through juxtacrine signaling (32, 33). It should be noted that once generated, PAF/ox-GPCs are rapidly metabolized by removal of the *sn-2* short-chained fatty acid by acetyl hydrolases. However, certain cell types such as keratinocytes have demonstrated the ability of PAF to exert its effects some distance away from the host cell (34). Previous studies by our group have shown that UVB-irradiated keratinocytes can induce the production of subcellular microvesicle particles (MVP), which contains PAFR agonist activity (34, 35).

Microvesicle particles are small membrane-bound particles that are shed from the plasma membrane of various cell types including keratinocytes (36). Their release is largely dependent on the action of the lipid enzyme acid sphingomyelinase (aSMase) (37, 38). Also named microvesicles and microparticles, MVPs are thought to provide a cellular mechanism to transport a variety of bioactive substances including proteins, lipids, cytokines, and nucleic acids (39, 40). Furthermore, their integral role within cell-to-cell signaling has suggested their mediation of pathogenic processes (41). Emerging literature suggests that UVB-generated ox-GPC PAFR agonists activate keratinocytic PAFR, which translocates aSMase to trigger MVP release from the plasma membrane (42). However, the exact mechanism linking UVB-irradiation with the cutaneous immune response and its pathological manifestations needs to be further elucidated. In this review, we highlight the potential role of UVB-generated MVPs and subsequent release of PAF in cutaneous immune responses.

## Background

### Cutaneous Microenvironment

Skin with subcutaneous fat (called the hypodermis) is the largest and most exposed organ of the human body. As such, it acts as a physical barrier, which leaves it vulnerable to a myriad of potentially harmful agents including bacteria, viruses and other environmental contaminants. In addition, the skin has important functions in regulating body temperature, preventing dehydration, and protecting against ultraviolet (UV) radiation (43). Cutaneous anatomy consists of an outer epidermal layer and an inner dermal layer that are joined by a basement membrane as well as an underlying layer of subcutaneous fat and connective tissue (44, 45). The epidermal layer is made up of five distinct layers that consist primarily of keratinocytes, which are bioactive cells that are highly proliferative and secrete a variety of cytokines (46). As keratinocytes detach from the basal layer of the epidermis, they stop dividing and undergo a final differentiation process known as cornification (44, 47). Melanocytes, Langerhans cells (LC), and Merkel cells are other cell types in the epidermis that are involved in melanin synthesis, immunoregulation and sensory functions respectively (46). The underlying dermis supplies nutrients to the epidermis and consists of two layers: the upper papillary layer and the lower reticular layer. The papillary dermis consists of densely packed fibroblasts whereas the reticular layer has lesser numbers of cells such as fibroblasts but contains more densely packed collagen and sits just above the subcutaneous fat (44). Various immune cells reside in the dermis which provide routine immune surveillance; these include macrophages, dendritic cells, T cells, mast cells, neutrophils and eosinophils. Inflammation and immune responses lead to significant proliferation of dermal immune cell populations (48, 49). The underlying hypodermis is a layer of subcutaneous tissue, composed of blood vessels and adipocytes that provide storage of lipids and fatty acids. Adipocytes help generate a number of bioactive lipid mediators and peptide hormones that partake in a number of dermal biological processes including cell signaling, inflammation, and regulation of local and systemic effects (50, 51). The connection between the local and systemic pathways are based on both internal and external variables including serotonergic, melatoninergic, and cholinergic pathways (52). The skin responds distinctly depending on the type of stressor and amount of exposure, which results in specific physiologic responses and subsequent signaling pathways. Of particular interest is the effect of UVB radiation on the skin and its effects on keratinocytes.

Ultraviolet radiation is implicated in a number of cutaneous pathologies and is divided into three distinct wavelengths: UVA (320-400 nm), UVB (290-320 nm) and UVC (100 -290 nm). Accounting for only 0.3% of the total light, UVB is absorbed by the epidermis, and can directly damage DNA by forming photoproducts including cyclobutane pyrimidine dimers and 6-4 photoproducts (53). In addition, UVB exposure results in the upregulation and release of various factors from keratinocytes including growth factors, antimicrobial peptides (human β-defensin-2, -3, ribonuclease-7 and psoriasin (SA100A7)) and cytokines (IL-1α, IL-1β, IL-6, IL-8, granulocyte colony stimulating factor [G-CSF], macrophage-CSF, interferon gamma [INF-γ], platelet-derived growth factor (PDGF) as well as the increased accumulation of reactive oxygen species (ROS) (54, 55). UVB also has additional roles in immune function by upregulating toll-like receptors and can disrupt the delicate skin microbiome (56, 57). One such consequence of prolonged UV-exposure is immune suppression, which includes inhibition of antigen presentation, induction of leukocyte apoptosis, and generation of immunosuppressive cytokines and (16). Additionally, UV-induced immunosuppression has been implicated to act through several molecular including DNA and membrane lipids, tryptophan in skin cells, and trans-urocanic acid (UCA) as well as through the depletion of other molecules including nicotinamide adenine dinucleotide (NAD) levels in keratinocytes (58). Moreover, UVB appears to inhibit immune reactions in an antigen-specific fashion, suppressing primary immune reactions. This is evidenced by suppressed contact hypersensitivity and delayed type hypersensitivity (DTH) reactions after UVB-irradiation, which show a diminished T-cell mediated immune response and generation of antigen-specific tolerance and desensitization (59–61). Extensive UVB damage alters vitamin D uptake, induces wrinkling of the skin, promotes photoaging, and can result in skin carcinogenesis as well as immunosuppression (14, 58, 62, 63). Simultaneously, vitamin D has been shown to have photoprotective effects against UV damage, which include diminishing the production of free radicals and attenuating DNA repair (64). As a result, the mechanisms by which UVB-mediated damage has a wide spectrum of effects in the epidermis and beyond are becoming increasingly important. Interestingly, UV radiation is able to upregulate local neuroendocrine axes through locally induced cytokines, corticotropin-releasing hormones, urocortin’s, proopiomelanocortin-peptides, and enkephalins. These local compounds can induce systemic effects such as the activation of central hypothalamic-pituitary-adrenal axis as well as immunosuppression (65). Of note, UVB enhances the production of a variety of bioactive lipids including PAF and eicosanoids such as PGE_2_, which appear to play an important role in UV-induced immunosuppression, inflammation, and carcinogenesis (26, 66).

### The Platelet-Activating Factor Family

In 1972, the term Platelet-activating Factor was first utilized in reference to a released product of IgE-induced basophil activation which results in subsequent platelet aggregation in rabbit models (67). Since its initial discovery, further investigations have indicated that PAF asserts a multitude of physiological and pathophysiological effects through binding with a specific G-protein-coupled receptor, PAF-R, extensively expressed by many immune and epithelial cell types (68). Despite new understandings of the functional significance, Benveniste and colleague’s cognomination persisted. The PAF family has been implicated in a variety of conditions ranging from malignancies to neurological conditions. PAF-related mechanisms have also been described in asthma and of interest to our group, UVB-mediated responses (42). Interestingly, PAF’s proposed acute and chronic impacts appear contradictory, the former pro-inflammatory and the latter immunosuppressive as will be discussed further (31).

**Figure 1:**
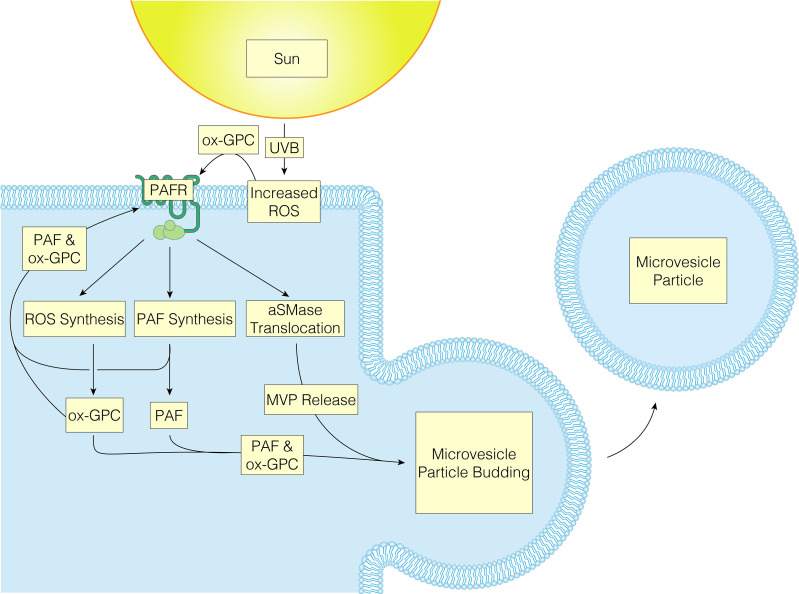
PAF biosynthesis pathways.

Throughout the body, PAF production can be stimulated by a variety of cell types. Through a variety of mechanisms, eosinophils, neutrophils, macrophages, monocytes, basophils, and mast cells all play a role in PAF synthesis (69). The biosynthesis of PAF has two separate pathways: a remodeling pathway and a *de novo* pathway (**Figure 1**). An interesting facet of the remodeling pathway of PAF production includes its influences from cell-specific inflammatory tracks. Upon cellular stimulation, increased intracellular calcium levels activate Ca-dependent MAPK kinase which phosphorylates phospholipase A_2_ (PLA_2_). PLA_2_ then deacylates alkylacyl-glycerophosphocholine to produce lyso-PAF and the unsaturated *sn-2* fatty acid, often arachidonate. Lyso-PAF (1-alkyl-sn-glycero-3-phosphocholine) is further acetylated by CoA lyso PAF acetyltransferase (LPCAT2) forming PAF (1-*O*-alkyl-2-acetyl-sn-glycero-3-phosphocholine) and Coenzyme A. The terminal enzyme in PAF biosynthesis, LPCAT2, is also highly expressed in inflammatory cells (16, 42, 70). One example of this includes its role in macrophages. Following lipopolysaccharides (LPS) binding to TLR4 on macrophages, protein kinase 2 activates MAPK which phosphorylates LPCAT2 resulting in a rapid surge in PAF. In addition, the PAFR, once activated, results in the activation of protein kinase Cα which phosphorylates LPCAT2, modeling a positive feed forward loop for PAF formation (16, 31, 69). Moreover, the *de novo* model of PAF production, unlike its counterpart, is not influenced by inflammatory pathways but plays an important role in baseline PAF levels due to constitutive activation. This pathway has three primary steps. Initially, 1-alkyl-2-lyso-*sn*-glycero-3-P is acetylated by CoA-alkyl-lysoglycero-P acetyltransferase forming 1-alkyl-2-acetyl-sn-glycero-3-P. Next, this intermediate undergoes dephosphorylation *via* an alkylacylglycerol-P phosphohydrolase forming 1-alkyl-2-lyso-sn-glycerol. Lastly, this glycerol molecule receives a phosphate group and choline by CDP-choline alkylacetylglycerol cholinephosphotransferase (25, 71, 72).

The metabolism of PAF is highly regulated by the PAF acetylhydrolase (PAF-AH) subclass of phospholipase A2 enzymes (73). By hydrolyzing the short-chained fatty acid moiety (e.g., acetyl group) at the *sn-2* position of PAF and related GPC, these signaling molecules form the biologically inactive lyso-PAF. The PAF-AH family members PAF-AH type I, PAF-AH type II, and plasma PAF-AH are pertinent to PAF degradation (31, 74). While these enzymes were originally named due to their role in PAF metabolism, they play a multitude of physiological and pathophysiological roles which are further discussed in the literature (73). The intracellular PAH-AH type II heterotetrameric has also been found in both sebaceous glands and epidermal keratinocytes (73, 75). Due to the rapid degradation and tight regulation of PAF and its implication in a vast number of processes, there is a need to better understand the mechanisms which allow for the systemic effects of PAF. Keratinocyte-derived MVPs are a major point of interest for this role.

### Microvesicle Particles

UVB-mediated bioactive product release can take place in a variety of ways, including through the release of MVPs. MVPs are extracellular vesicles that are formed from outward protrusion of the plasma membrane when there is an increase in intracellular calcium (76). Ranging from 100 – 1,000 nm, MVPs are important for cellular signaling due to them carrying a variety of different proteins, lipids, and mRNA. MVPs can be shed during physiological conditions including cell growth but also are increased in a variety of pathological processes ranging from hypoxia to oxidative stress (40, 77). Many cytokines including TNFα and cell damage can release MVP (38). They are present in a range of physiologic and pathologic processes throughout the body (77–79). Of particular interest are MVPs generated by a keratinocyte cell line through budding from the plasma membrane which contains PAFR agonist activity (80, 81). Both MVPs and PAF are released due to activation of PAFR. In fact, MVP production and activation can be triggered by a great variety of mechanisms, including acute alcohol poisoning (35, 82).

Throughout the human body, extracellular vesicles like MVPs and smaller exosomes frequent matrices of the system. UVB has been demonstrated to induce the release of both of these particles (34, 83). While MVPs are often parceled with exosomes, each are separate entities expressing different characteristics. Exosome formation occurs *via* reverse budding of multivesicular bodies prior to cellular release (84, 85). This process allows for delivery of exosome contents with plasma membrane fusion (85). On the other hand, MVPs are contrived from external budding and fission of cellular plasma membrane which allows for an additional function in MVPs of cargo delivery into the extracellular environments (86). The variance in formation of each of these extracellular vesicles results in discrepancy in sizes as well. Exosomes typically range from 30nm – 150nm with less deviance from mean values (84–86). This range is smaller than the previously mentioned range for MVPs and has a lower peak value. Due to the external plasma membrane invagination formation mechanisms of MVPs, cytosolic elements are accrued secondary to proximity to the plasma membrane. In exosomes, contiguity with intraluminal vesicles play a greater significance in free exosome content (85, 87).

While the exact mechanisms of keratinocyte-derived MVP generation and release are unclear, much research has been done on the biogenesis of MVPs (34). The production of MVPs has three elemental steps. To begin, membrane and lipid proteins are reorganized into distinct groups within the plasma membrane. Next, these microdomains aid in various pathways attracting cargo. Lastly, in conjunction with other machinery, membrane budding and fission is promoted (88). The initiation of these processes is highly unlikely to be secondary to spontaneous intrinsic property changes of the phospholipid bilayer but rather unclear emerging external mechanisms (86). In particular, MVP generation from diverse stimuli involve the enzyme acid sphingomyelinase (aSMase) appears to play an important role in this process (42). Furthermore, MVP cargo, which is dependent upon parent cell archetype, is preferentially gathered through a variety of possible mechanisms- most notably ARF6 of the GTPase family (89). Downstream effects of MVPs are secondary to their respective cargo, highlighting the importance of these pathways. Of interest, keratinocyte-formed MVPs contain a myriad of bioactive agents and molecules including PAF and ox-GPC, which have implications in systemic immune responses (34). Of interest, protein cytokines found in UVB-generated MVP contain lesser numbers of classic pro-inflammatory cytokines (e.g., TNF-alpha), but increased numbers of anti-inflammatory cytokines such as IL-1 receptor antagonist, in comparison to baseline (unstimulated) MVP (37).

As previously discussed, MVPs functions are dependent upon the appropriate intercellular messaging and respective cargo. As more research is needed on keratinocyte-derived MVPs uptake at their respective target cells, we will only briefly cover this topic. Parent cells of MVP, by nature of their biosynthesis pathway, additionally impact the plasma membrane content of this extracellular vesicle. The adoption of plasma membrane from origin cells is believed to play a role in MVP intercellular communication (88). With this in mind, certain MVPs transmit signals by direct physical contact between membrane-associated ligands and cell surface receptors. One such example is the production of chemokine (C-C motif) ligand 3 (CCL3), chemokine (C-C motif) ligand 7 (CCL7) and IL-24 in T-cell-derived MVPs co-cultured with *in-vitro* keratinocytes (90, 91). Indirect mechanisms of MVP function have also been observed. For instance, MVP release of cargo into extracellular milieu near the vicinity of target cells has been shown to stimulate cell surface receptors (92). Furthermore, MVPs can merge with beneficiary cells’ plasma membranes, sending internal cargo into the recipient cells’ cytoplasm with the additional potential of membrane property transference (93). Finally, select MVPs are absorbed into their destination cells through non-selective macropinocytosis or endocytosis (94).

## UVB Activation of MVPs

UVB radiation (290-320 nm) results in a pro-oxidative stress that exerts significant effects within the skin. One of which is the generation of ROS, which modify biologically active agents, such as lipids (28). GPC is an integral structural lipid found in all cellular membranes and functions as a precursor of bioactive PAF. Due to the presence of bisallylic double bonds, esterified polyunsaturated fatty acids are vulnerable to oxidation from ROS attack of the hydrogen donors. This results in the introduction of oxy functions to the chain of carbon atoms, rearranges bonds, fragments carbon-carbon bonds by β-scission, which can all give rise to a multitude of lipid reaction products (27, 28). In particular are a series of phospholipids with oxidatively fragmented *sn-2* acyl residues with a terminal methyl group or ω-oxy function. Potent PAF agonists (ox-GPC) are generated if the oxidatively modified *sn-2* acyl residue is a 1-alkyl GPC (26–28). There are multiple literature reports that link UVB generation of ROS with subsequent activation of the PAF system (10, 16, 17, 23, 29, 30). Early research into these findings was based on a knowledge of how UV response affects the cell membrane rather than the nucleus (95). Additional evidence found overexpressing the PAFR enhances PAF synthesis by keratinocytes in response to UVB radiation, while administering a PAFR antagonist inhibits PAF synthesis in UVB radiated keratinocytes (96). Furthermore, Yao and colleagues discovered that a PAFR antagonist inhibited UVB-mediated skin inflammation and TNF-alpha synthesis in the photosensitive *Xpa-/-* mice (29). Interestingly, it has also been found that PAFR expression is necessary for UVB-induced activation and subsequent migration of mast cells (97). Thus, UVB acts as a pro-oxidative stressor that generates ox-GPC and subsequent activation of PAFR through its PAFR agonist activity. Of importance, ox-GPC activation of the PAFR can generate enzymatic PAF synthesis, thus forming a positive feedback loop (31).

**Figure 2:**
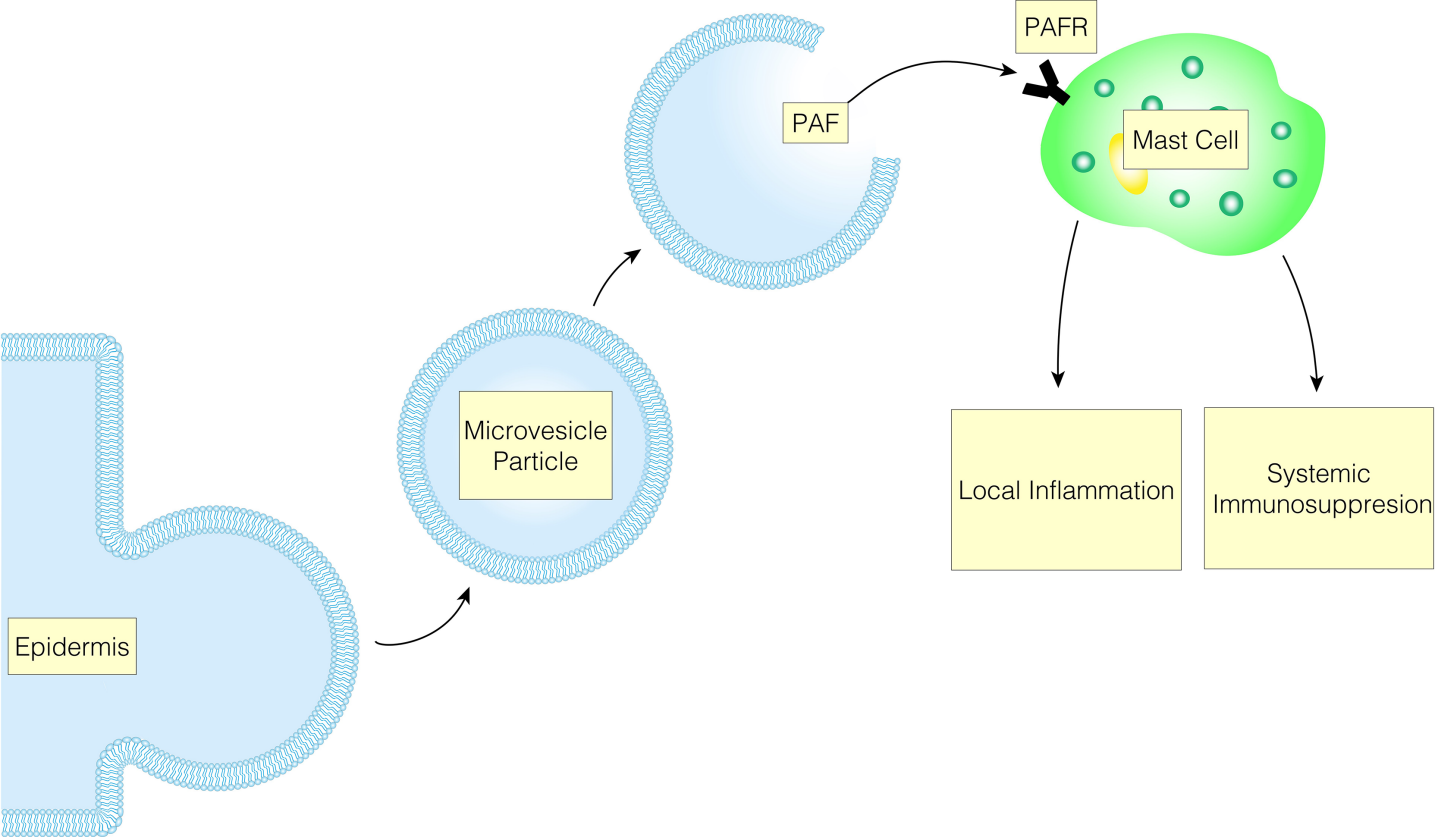
UVB oxidized-GPC results in PAFR activation, causing MVP to be released from the keratinocyte plasma membrane, allowing PAFR agonist to be transported to additional locations.

UVB radiation and PAFR activation of keratinocytes have been noted to result in multiple similar signaling pathways, implying that some UVB-induced effects are mediated *via* PAFR or modified by associated PAFR activation (19, 20). There are several cutaneous cell types that express PAFR including keratinocytes, mast cells, monocytes, granulocytes, and B cells (30, 97–102). The actions of PAF and its agonists are mediated through the PAFR, a unique G-protein-coupled seven transmembrane receptor (GPCR), which when activated results in several intracellular signaling pathways (68). These include mitogen kinase (both ERK and P38), JNK, and can also indirectly activate the EGFR (80). While coupled to G proteins Gi, Gq, and G12/13, PAFR also controls the production of inositol 1,4,5-triphosphate (IP_3_) and calcium mobilization in GPCRs, while suppressing forskolin-stimulated cAMP synthesis (103). Emerging literature supports the theory that UVB produces ox-GPC PAFR agonists, which then act upon PAFR-positive keratinocytes, resulting in PAFR activation. As a result of PAFR activation, more PAF is produced enzymatically as well as more ROS and subsequent ox-GPC. Finally, PAFR activation induces aSMase to translocate to the plasma membrane causing MVP to be generated and released from the keratinocyte plasma membrane. As the PAF/ox-GPC will be retained in the plasma membranes this allows PAFR agonist to be transported in the MVP to additional locations (**Figure 2**) (42). Of interest, studies of the crystal structure of the PAFR have revealed one of the protein loops appears draped over the central area of this GPCR (100). This novel putative conformational change suggests that PAFR agonists interact with the receptor optimally from the plasma membrane rather from the exterior of the cell. Hence, it is possible that the PAFR agonists in the MVP when are incorporated into the target cell could be in a pharmacologically active form. In sum, a feed-forward loop is created because PAFR activation results in continual release of PAF from MVPs and subsequent activation of PAFR.

As mentioned previously, a common lipid pathway causing MVP release is the stimulus-mediated translocation of the enzyme aSMase from lysosomes to plasma membranes (38, 42, 104). Importantly, activation of PAFR has been shown to generate membrane translocation of aSMase as well as boost its enzymatic activity (105, 106). Multiple lines of evidence support aSMase serving as the effector for PAFR-mediated MVP release. For instance, our group found that both application of the metabolically stable PAFR agonist carbamyl-PAF (CPAF) as well as UVB exposure led to increased aSMase enzymatic activity in HaCaT keratinocytes (37). In addition, after UVB irradiation, application of the aSMase inhibitor imipramine inhibited MVP generation in HaCaT keratinocyte cells, human skin explants, and murine skin. Of note, multiple agents including many tricyclic anti-depressants act as functional inhibitors of aSMase (FIASM) (107).These findings were further substantiated by the use of PAFR-KO (*Ptafr-/-*) and aSMase-KO (*Smpd1-/-*) mice, which corroborated functions of PAFR and aSMase in UVB-mediated MVP release in skin (37). Of note, application of the phorbol ester phorbol-12-myristate -13-acetate (TPA) results in MVP generation in PAFR-deficient but not in aSMase-deficient hosts. Finally, topical treatment with the aSMase product C2 ceramide also resulted in amplified MVP levels in mice, including aSMase-deficient animals. Ultimately, UVB-induced production of MVPs relies on a process that involves both PAFR signaling and aSMase.

There are many stressors and pathways that can generate MVP. However, our group was the first to demonstrate the ability of UVB to induce PAF-mediated MVP formation and release (35). Using a keratinocyte-derived cell line (HaCaT), UVB irradiation resulted in increased levels of MVP generation. Furthermore, application of the non-metabolizable PAFR agonist CPAF resulted in MVP formation in a dose-dependent manner. In addition, to test if the PAF system was involved in UVB-mediated MVP generation, we exposed UVB to PAFR-positive and PAFR-negative cell lines (KBP and KBM, respectively). UVB-irradiation generated MVP formation in only the PAFR-positive cell line, while no differences in MVP levels were seen in the PAFR-negative cells. Similar findings were noted using PAFR-deficient mice. These findings demonstrate PAF’s involvement in UVB-induced MVP formation, which adds to the extensive literature supporting the role of the PAF system in mediated UVB-induced acute inflammation and delayed immunosuppression (19, 27, 66). It is also known that UVB-induced PAF agonists involve ROS that can be blocked by antioxidants (18, 27, 66). Our group found that UVB-mediated MVP generation involves ROS-induced PAF agonists, as evidenced by the ability of antioxidants to block MVP production (35). More specifically, pretreatment of HaCaT cells with N-acetylcysteine and vitamin C resulted in hampered UVB-induced MVP release while CPAF-mediated MVP release was unaffected. Furthermore, the UVB fluences required to produce MVP in keratinocytes are quite high, with a two- to three-fold increase in the minimum erythema dosage required to detect quantifiable MVP within the skin (108). While addressing this phenomenon, our group demonstrated that pretreatment of epithelial skin cells with PAFR agonist can synergize with low fluences of UVB to produce high levels of MVP. This suggests that MVP could play a role in combinatorial pathologic processes involving UVB, which is important since synergistic responses from multiple agents are likely more common than presently recognized (108).

PAF persists in the cellular membrane after production where it can operate on itself or nearby cells *via* juxtacrine signaling (32, 33). Additionally, studies have shown that in certain cell types, such as keratinocytes, PAF can exert its effects some distance away from the host cell (34). This phenomenon is likely the result of MVPs, which are thought to provide PAF protection from degradation by PAF-AH as opposed to being free or protein-bound within tissue fluids (42). By extension, the advantage of this arrangement is the protection of metabolically unstable compounds from enzymatic degradation. Thus, UVB-induced systemic effects may be mediated in part by MVPs produced by epithelial cells.

## MVP-Mediated Local Inflammation

UVB damage causes both local and systemic inflammation and immunosuppression. While considering the cutaneous response to environmental stressors, there are a number of cytokines and cells signaling pathways that are mediated through T-cells, antigen-presenting cells (APCs), mast cells, fibroblasts, and keratinocytes. Consequently, the release of many cytokines including TNF-alpha, IL-6, IL-8, IL-10, and others leading to a range of pathological processes (11, 109). The aforementioned effector cells and cytokines create a complex interaction that initially generates an acute and local reaction that may eventually progress to a systemic response. It appears that this UVB-induced systemic response is in part mediated by MVPs, which act on these effector cells and amplify the UVB-damage. The end result is an MVP-mediated inflammation and immunosuppression that may give rise to a number of pathological processes.

As aforementioned, UVB produces ox-GPC PAFR agonists, which then activate PAFR in PAFR-positive keratinocytes. More PAF is created enzymatically as a result of PAFR activation along with more ROS and hence more ox-GPC. Moreover, its activation also causes aSMase to translocate, leading to MVP release. The epidermis then releases MVP, which contains PAF and ox-GPC (42). Locally, a variety of downstream effects are then generated as previously mentioned (11, 109). Recently, MVP release has been found to be associated with the production of the pro-inflammatory cytokine IL-8. Studies done by Bhadri and colleagues explored the possible link between the pro-inflammatory cytokine IL-8 and MVP. The authors demonstrated that pretreatment of HaCaT keratinocytes with PAFR agonists synergizes with low fluences of UBV to produce increased levels of IL-8 and MVPs (110). Further illustrating this linkage, application of the FIASM imipramine blocked both MVP and IL-8 release following UVB treatment (110). Furthermore, the ability of IL-8 to activate and attract neutrophils suggests that it may play a role in the acute UVB response. This is highlighted by the report that treatment with an IL-8 neutralizing antibody, which reduced UVB-induced synthesis of fibroblast neprilysin and matrix metalloproteinase 1 (MMP-1) in keratinocyte-fibroblast cocultures (94). Interestingly, the role of PAF in local immunosuppression is still under debate due to Sahu and colleagues showing that UVB-mediated LC depletion is not mediated though PAFR (111). Rather, UVB-exposed mouse skin treated with the contact allergen DNFB (2,4-dinitro-1-fluorobenzene) resulted in a significant inhibition of contact hypersensitive response in both WT and PAFR knockout mice. Additionally, the presence of LC was reduced in both types of mice compared to their sham irradiated controls (111). Thus, it appears that MVP-mediated PAF release caused by UVB needs further research to establish a causal role in the local immune response.

**Figure 3:**
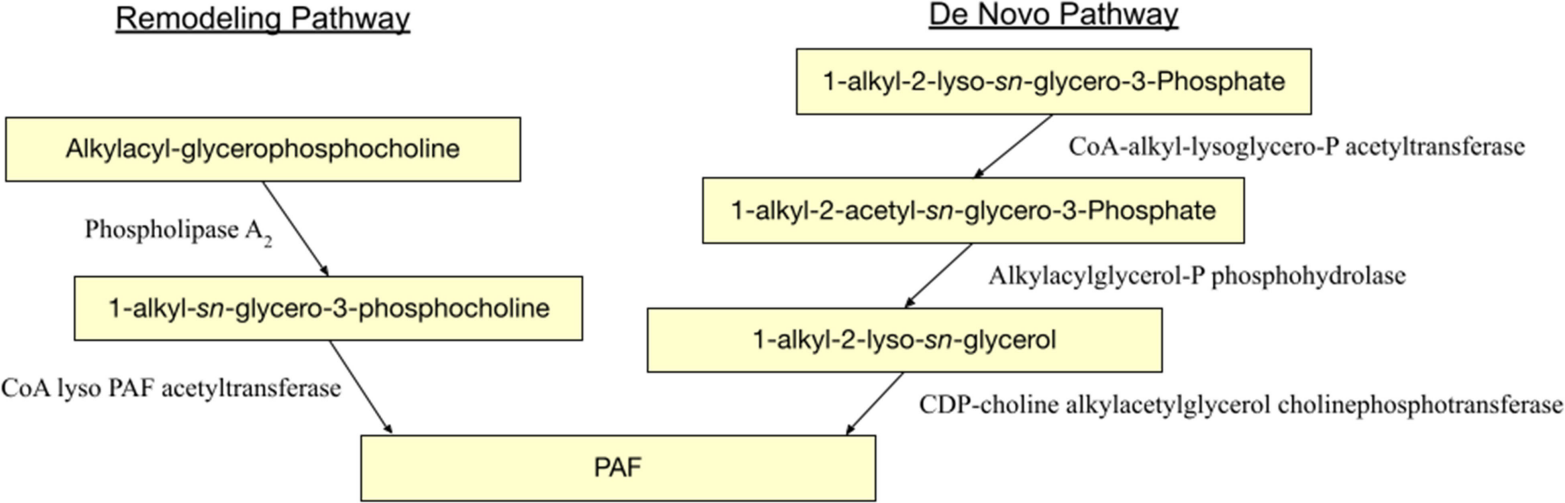
UVB-induced MVP release leads to mast cell PAFR activation, which results in immunological responses characterized by both local and systemic inflammation and immunosuppression.

The immunological responses to UVB are characterized by both local and systemic inflammation and immunosuppression. These phenomena are not mutually exclusive as many acute mediators serve to bridge the local and systemic responses. The connection between these two systems is illustrated by the effect of PAF on keratinocytes with the mast cell playing an effector role (**Figure 3**). UVB-induced damage mediated through PAF on keratinocytes decreases DNA repair mechanisms by decreasing the expression of response elements such as ataxia telangiectasia and rad3 related protein (ATR) (112). Additionally, the secretion of PAF by keratinocytes increases IL-10, activates COX-2 and decreases delayed type hypersensitivity reactions (102, 113). Furthermore, PAF release from keratinocytes promotes mast cell migration into the lymph nodes and subsequent systemic effects. Moreover, PAF stimulation of mast cells upregulates the epigenome to increase the expression of DNMT1/3b and p300, and decreases the expression of HDAC2. Ultimately, this increases the responsiveness of mast cells to CXCR4 agonist, in which mast cells will release IL-10 and histamine (112, 114, 115). Further, this relationship is supported by UV radiation not suppressing the contact hypersensitivity (CHS) response in mast-cell deficient mice, but when these mice are reconstituted with normal bone marrow-derived mast cells, UV-induced suppression of CHS is restored (115).

The role of LC UV-induced PAF-mediated local inflammation is still being established, but there appears to be a relationship between PAF-induced LC migration, consequently leading to immunosuppression (116). In particular, UV irradiation induces expression of RANK-L in keratinocytes which stimulates the migration of LC to the lymph nodes where they activate immunological Treg cells, decreasing the DTH, CHS, and tumor immunity (117–120). PAF-induced PGE2 may be implicated in the process by upregulation of RANK-L on keratinocytes since inhibiting PGE2 binding to its receptor EP4 with specific receptor antagonists inhibits RANK-L upregulation, impairs UV-induced Treg activation, and inhibits UV-induced CHS suppression (121). PAF is also known to induce regulatory dendritic cells through several cytokines including PGE2 and IL-10 (122). These examples help illustrate the growing picture linking UV exposure, MVP-mediated PAF release, and subsequent local and systemic immunosuppression.

The relationship between UVB-mediated MVP release in systemic cutaneous disease is still evolving, but current literature has shown the PAF system induces inflammation in a variety of skin pathologies. For example, PAF can be found to be increased in inflammatory lesions of psoriasis favoring Th17 development. More specifically, this appears to occur through PAF-mediated increase in the expression of IL-1ß, IL-5, and IL-23 in LC and keratinocyte, subsequently leading to Th17 development (123–125). Other illustrations of the detection of the PAF in cutaneous disease states are in bullous pemphigoid, sunburn, cold urticaria and in burn injuries (66, 126–128). However, it is unclear in these pathogenic states if the PAF measured is truly contributory or serves as a “bystander effect” of the concomitant inflammation. In sum, there is a need to use specific pharmacologic and genetic tools to expand on this body of knowledge, particularly MVP’s role in UVB-induced cutaneous inflammation, immunosuppression, and systemic cutaneous disease.

## Delayed Immunosuppression and Blocking of Tumor Immunity

### Tumorigenesis/Metastasis in Melanoma

Exposure to UVB radiation is a major risk factor in developing skin cancer, especially melanoma. UVB induces oxidative stress and generates lipid mediators such as PAF and Ox-GPC (129). These factors signal inflammatory cytokines that lead to systemic immunosuppression, which leads to tumor augmentation in experimental murine models by two-fold (130). Not only are MVPs released by UVB-induced PAFs, Lima et al. demonstrated that malignant melanoma cells (B16F10) produce a large quantity of microvesicles *in vitro* that mediate immunosuppression and tumor progression (131). TGF-beta is an important immunoregulatory factor produced by phagocytes; B16F10-produced MVP upregulates this production, leading to changes in the tumor microenvironment promoting tumor dissemination (131). Moreover, it is noteworthy that antioxidants attenuate the effect of UVB-irradiation induced melanoma growth, suggesting that reactive oxygen species (ROS) leading to PAF release could play an important role in tumor growth. Likewise, UVB radiation activates tumorigenesis through the PAF-PAFR pathway. While PAFR are not found in melanocytes, the release of PAFR agonist due to environmental stressors, such as UVB, activates a cascade of downstream effects that leads to tumorigenesis and metastasis. Overexpression of PAFR has been shown to lead to acanthosis and increased numbers of dermal melanocytes, fibroblasts, mast cells, filaggrin and endothelial cells (132, 133). Interestingly, PAFR transgenic mice spontaneously developed hyperpigmentation on the ears, tails, and external genital area and melanocytic tumors in the dermis with age (132). It is likely that keratinocyte hyperplasia stimulated this growth of dermal melanocytes directly or indirectly. Of note, topical use of PAFR antagonist, WEB 2086, inhibited keratinocyte proliferation in both transgenic mice and control mice, suggesting that PAFR inhibition may mediate cellular hyperplasia and tumor growth (132). However, the phenotype of the PAFR transgenic mouse could likely be due to inappropriate expression of the PAFR using a widespread actin promoter. Sahu and colleagues found the mechanism which leads to melanoma tumor progression is due to the systemic expression and activation of PAFR; and that whether the tumor cells themselves expressed PAFR or not does not impact the tumor development, suggesting that PAFR-mediated anti-tumoral immunity effects are involved (130). Support for this finding relies on several mechanisms. One, PAF-induced release of inflammatory cytokines, such as IL-10, Treg, IL-1, and TNF-alpha lead to systemic immunosuppression and tumor metastasis (130, 134). Two, UVB-induced immunosuppression can be blocked by PAFR-antagonists, therefore attenuating the effect on tumorigenesis (135). Three, administration of a systemic PAFR agonist, carbamoyl PAF (CPAF), will increase tumor growth in wildtype mice or mice injected with PAFR onto host cells. However, CPAF failed to augment the tumor size in *PAFR -/-* mice (130). Lastly, PAFR acetylhydrolase (PAFR-AH), an enzyme that breaks down PAF, will decrease tumor vascularization and growth (133). These observations suggest that systemic PAF/PAFR expression and activation contribute to carcinogenesis, and inhibition of these reactions may provide insight to cancer treatment.

Melanoma is the most lethal skin cancer due to its high likelihood to metastasize and ability to evade therapy (136). The PAF-PAFR pathway has been implicated for the promotion of metastasis in melanoma (134, 137, 138). An inflammatory tumor microenvironment leads to proliferation, invasion, and angiogenesis of tumors which may metastasize. Matrix metalloproteinase-2 (MMP-2) is a main contributor to cancer cell migration, which can be stimulated by PAFR (139). Melnikova and their group studied melanoma metastasis to the lung and found that these cell lines all expressed PAFR to a varying degree, although the extent of metastasis does not correspond to the level of PAFR expressed (5). PAF upregulates multiple pathways that lead to cell migration and metastasis (137, 138). Importantly, PAF-induced expression of pro-MMP-2 was related to PAF-induced cAMP response element (CREB) and activating transcription factor (ATF-1) phosphorylation, and addition of a PAFR antagonist prevented this stimulation (137). It is thought that PAFR-induced CREB phosphorylation in more aggressive melanoma is through two intermediate signaling proteins, PKA and p38 MAPK. In addition, incubation of melanoma cell lines with the PAF analog, CPAF, increased phosphorylation of CREB (138). Another study looking at lung metastasis relating to melanoma also found that PAF enhances metastasis (134). In Im and colleagues’ experiments, pulmonary metastasis from B16F10 melanoma were significantly increased with addition of dose- and time-dependent PAF. Furthermore, administration of PAF-R antagonists reduced the pulmonary tumor colonization (134). Additionally, IL-1 and TNF-alpha were thought to be at least partially mediated by PAF generation, which can cause metastasis (134). Another important mechanism for metastasis is the cooperation between PAR-1 and PAFR to regulate the expression of MCAM/MUC18 (melanoma cellular adhesion molecules) (138). Taken together, metastatic melanoma carries higher expression of PAF/PAFR and increases migration through mechanisms modulated by the inflammatory tumor microenvironment.

Angiogenesis is an important step in tumor metastasis. Platelets and factors in the coagulation cascade, especially thrombin, are stimulated by PAFR and play crucial roles in vascularization that leads to tumor growth and metastasis (137, 138). Notably, PAF/PAFR modulate tumor cell adhesion to endothelial cells, angiogenesis, tumor growth, and metastasis (137). Inhibition of neoangiogenesis by inhibiting PAF could play a part in mediating metastasis. Indeed, mice bearing B16F10/PAF-AH tumors survived significantly longer than those bearing B16F10/neo tumors (133). Thus, it is clear that the PAF/PAFR systemic activation is implicated in tumorigenesis, which further highlights the need to understand how UVB-induced MVPs mediate this process. Finally, metastatic melanoma has especially poor prognosis due to failure of response to chemotherapeutics and radiation therapy. Therefore, understanding mechanisms for metastasis and tumor survival will provide insight to better treatment in melanoma.

### Chemotherapeutics

Environmental stressors, such as UVB radiation and cigarette smoke, could help tumors such as melanomas escape antitumor immunity. Similarly, chemotherapy and radiation therapy increase PAFR expression in tumors as a protective response through generating reactive oxygen species (ROS). ROS activates host-immunity and increases glycerophosphocholine (GPC) species that can act as PAFR agonists (129, 130, 140). The release of PAF subsequently augments cytokine production through the NF-kB pathway, assisting the evasion of chemotherapy. The NF-κB pathway leads to the loss of E-cadherin, contributing to melanoma invasion and resistance to apoptosis (136). Another study showed that cancer therapy activated PAF-R systems augment the cytokine production, specifically IL-8 and TNF-alpha through the NF-κB pathway (141). Cyclooxygenase-2 (COX-2) is a major promoter in immune suppression in melanoma, through its ability to stimulate angiogenesis, inhibit apoptosis, increase cellular proliferation and increase cellular invasiveness, enhance immunosuppression, and produce mutagens (136). PAF is deducted to be a component of oxidative stress and these downstream pathways, and manipulation of PAF/PAFR gene expression, provides an understanding of tumor cell reactions to ROS and chemotherapy.

Cell repopulation is the phenomenon where tumor cells that survive chemotherapy or radiation therapy will undergo accelerated proliferation (142). Thus, finding the mediator that allows tumor cell survival will improve cancer treatment outcomes. Treatment with cisplatin increased PAFR gene expression and accumulation of its products in SKmel37 (melanoma cells) *in vitro* that express PAFR (143). Blocking the release of PAF induced by chemotherapy or the PAF systemic effect may enhance cancer treatment. Indeed, Onuchic et al. found a combination therapy with cisplatin and WEB2086, a PAFR-antagonist, in melanoma-bearing mice showed slowing tumor progression and increasing tumor regression (143). It is conceivable that PAF release protects tumor cells. Another example in the literature was done by Sahu et al. with the focus on ROS and COX-2 inhibition. Both chemotherapy and radiation therapy induce the release of PAF and PAF-like ox-GPC due to oxidative stress and generation of ROS. In a murine model of melanoma, addition of antioxidant regimen reduced the formation of PAFR agonist activity, therefore preventing the augmentation of tumor growth (130). Moreover, COX-2 inhibitors contribute to antitumor immunity by downregulating IL-10 and Tregs that modulate the systemic effect of PAFR-mediated tumor growth (130). Inhibiting effects of PAFR will block immunosuppression and photocarcinogenesis as well as accelerating DNA repair. Therefore, PAF induced immunosuppression may promote survival response in tumor cells (144). Finally, PAF antagonists may work synergistically with other agents that reduce immune suppression, such as 5-HT_2A_ receptor antagonists. Evidence shows that combination of PAF and 5-HT_2A_ receptor antagonists blocks skin cancer induction in UV-irradiated mice (145). These antagonists first prevent UV-induced damage in the skin from apoptosis after a single exposure to UV radiation, then prevent cytokine release and immune suppression (145). In conclusion, these results provide compelling evidence that potentially future chemotherapeutics could be created by manipulating the PAF/PAFR-mediated responses.

### Prosurvival Response in Tumor Cells - Anti-Apoptosis

Most chemotherapeutic agents exert their therapeutic effects through programmed cell death. It is understood that some cancer treatment failures can be attributed to tumor cells’ prosurvival response mechanisms that escape the apoptotic activities induced by chemotherapy. Chemotherapy and radiation therapy cause oxidative stress and induce PAF/PAF-like molecules release. PAFR-activated modification of cell microenvironment and upregulation of anti-apoptotic molecules favor tumor growth (146, 147). Specifically, PAF increases the expression of mRNA and protein synthesis of anti-apoptotic factors and inhibits caspase activities (147). Tumor-derived microvesicle particles are another source of anti-apoptosis endorsed by cancer cells to evade cell death (148). Chemotherapeutic agents induce cell death by increasing mitochondrial enzyme caspases. De Olivera and colleagues found that combination of dacarbazine (DTIC) and WEB 2170 (PAFR antagonist) significantly improves survival of B16F10 melanoma-bearing mice compared to either agent used alone (146). Using a PAFR antagonist alone, however, did substantially delay tumor growth (146). PAF molecules are found to inhibit the activities of caspase-3, caspase-8, and caspase-9, as well as cell death induced by etoposide, a common chemotherapy agent (147). These findings suggest that tumor cells release PAF/PAF-like molecules to prolong tumor survival and escape treatment desired cell death.

While abundant reports provide evidence that PAF leads to anti-apoptotic activities, some have found that the opposite could also happen. PAF may produce pro-apoptotic response to chemotherapy as well as anti-apoptotic response depending on which agent is being used (149). This finding complicates our understanding of the effects PAF has on chemotherapeutics and survival of tumor cells. The differences in PAF effect on chemotherapy may be due to varying pathways that PAF can induce. Depending on how the cancer medication works and what it targets, PAF may augment or attenuate the efficacy of chemotherapeutics. PAFR, for example, can enhance apoptosis induced by etoposide and mitomycin C, but not by other agents such as C2 ceramide or tumor necrosis factor related apoptosis-induced ligand (TRAIL) (149).. In sum, further research is needed to study the specifics of how PAF/PAFR affects cancer treatment and tumor cell survival response.

## Mechanistic Insight Through Pharmacological Interventions

The downstream effects mediated by UVB-induced MVP release have been implicated in both local and systemic pathological processes, including inflammatory responses, immunosuppression, and blocking of tumor immunity. As outlined, the mechanism involves UVB-generated ox-PAFR agonists and subsequent MVP generation, which then transports several bioactive molecules such as PAF both locally and systemically. There are a number of pharmacological agents that may modulate this pathway including aSMase inhibitors, antioxidants, COX-2 inhibitors, and PAFR modulators (agonists and antagonists). These agents may serve as potential pharmaceutical strategies to mitigate the pathological consequences seen from UVB exposure.

### aSMase Inhibitors

MVP and ceramide release are dependent upon the lipid enzyme aSMase (38, 150). Thus, as evidenced by multiple lines of evidence, aSMase inhibitors pharmacologically inhibit the effects of MVPs and PAF. The literature demonstrating the utility of aSMase inhibitors within the skin is limited. However, there are many models supporting their value in other tissues and organ systems. For example, the mechanisms driving formation of hepatic steatosis are due in part to the activation of aSMase and the production of ceramide in response to ethanol consumption. Liangpunsakul and colleagues found that aSMase inhibitors, such as imipramine, may serve as a therapeutic target for alcohol-induced hepatic steatosis by inhibiting the release of ceramides by aSMase (151). In addition, transfusion-related acute lung injury (TRALI) is mediated by ceramide-mediated endothelial barrier dysfunction, in which extracellular vesicles (EVs) may be required for transport from platelets to endothelial cells. McVey and colleagues reported that blockage of aSMase reduced the formation of EVs and may present as a promising strategy for TRALI prevention (150). By increasing vascular permeability, PAF is a known mediator of pulmonary edema in acute lung injury. This is due in part through the activation of aSMase (152). Yang and collaborators found that pharmacological inhibition of the aSMase pathway blocked the PAF-induced increase in caveolin-1 and endothelial nitric oxide synthase, suggesting aSMase inhibitors as a novel mechanism to regulate vascular permeability (152). Importantly, Chauhan and colleagues explored the role of PAFR signaling in MVP release and the underlying mechanisms using non-small cell lung cancer cell lines. They found that aSMase inhibition significantly blocked MVP release, highlighting the utility of modulating the aSMase and PAFR pathways for targeted therapies in lung cancer cells (153). Lastly, the Sahu research group determined the significance of PAFR in chemotherapy-mediated MVP generation in human pancreatic cancer cells and found the inhibition of aSMase blocked the generation of MVP (154). The aforementioned studies demonstrate the utility of aSMase inhibition in prevention of various pathologies within targeted tissues.

While sparse, there is some literature highlighting the usefulness of aSMase inhibitors within the dermis. Nakatsuji and collaborators found that *C.acnes* Christie, Atkins, Munch-Peterson (CAMP) factor may hijack host aSMase to increase bacterial virulence in order to impair and invade host cells (155). Similarly, Ma and colleagues demonstrated how *S.aureus* α-toxin activates aSMase in macrophages and precipitates the release of ceramides, which activate the inflammasome and mediate the generation and release of cytokines (156). Of note, a-toxin has been reported to be a potent stimulus for enzymatic PAF production (151). These findings suggest that inhibition of aSMase may block cutaneous microbial-driven pathogenesis. Furthermore, UV irradiation is known to impart a variety of negative pathological consequences within the skin. Appelqvist and colleagues explored the initial signaling during UV-induced damage in human keratinocytes by investigating apoptosis induction and lysosomal exocytosis. The authors found that the addition of anti-aSMase reduced the activation of caspase-8, which plays a central role in the execution-phase of cellular apoptosis (157). Likewise, UV irradiation stimulates the generation of ceramide through the *de novo* synthesis and hydrolysis of sphingomyelin. Kim and collaborators found that UV-induced intracellular ceramide may activate matrix metalloproteinase-1 (MMP-1) expression in dermal fibroblasts *via* JAK1/STAT-1 pathway (158). The findings from these two reports suggest that the targeted modulation of ceramide signaling and aSMase may offer a novel therapeutic approach to allay the risk of UV-mediated cutaneous pathology. Similarly to aSMase inhibitors, antioxidants have shown utility in the prevention of MVP release.

### Antioxidants and PAFR Antagonists

The PAF/PAFR signaling pathway has been shown to be exploited by UVB-induced oxidation and PAFR-agonists. This allows for the therapeutic potential of pharmacological agents including antioxidants and PAFR antagonists. Antioxidants may diminish the effects of UVB-induced ROS, which generate PAF agonists and trigger MVP release (34). There are multiple lines of evidence exhibiting the ability of antioxidants to block UVB-mediated PAFR agonist formation and UVB-MVP release (27, 34, 35, 66, 130). Moreover, the antioxidants N-acetyl cysteine, 1,1,3,3-tetramethyl-2-thiourea, and vitamins C and E completely suppressed PAF synthesis in cultured keratinocytes and human skin (159, 160). Furthermore, Sahu and colleagues demonstrated that pretreatment with antioxidants could block PAFR-dependent tumor growth (129). Antioxidants also restricted gefitinib and erlotinib ROS generation and subsequent PAFR activation, which highlights how modulation of PAFR signaling can modify the cellular responses of targeted cancer therapies (153). Lastly, pretreatment with PAFR antagonists and the antioxidant vitamin E resulted in inhibition of UVB-induced TNF-alpha production, suggesting that epidermal PAF-R may be a pharmacological target for UVB in skin (19).

As aforementioned, PAF-R antagonist intervention has the potential to reduce the negative effects of PAFR agonist signaling. Early *in vitro* literature showed pretreatment of PAFR positive keratinocytes with PAFR antagonists Web 2086 and A-85783 reduced UVB-induced TNF-alpha production. Zhang and collaborators provided additional support of the role of PAFR antagonist in the skin, demonstrating that sebaceous glands express PAFRs and PAFR antagonism can suppress COX-2 production in sebaceous glands (161). Interestingly, PAFR activity has been inhibited by pharmacological inhibitors of protein kinase C (PKC), implying that PKC mediates some PAFR actions (162). Inasmuch the subsequent release of MVP particles leads to immunomodulatory effects, the role of PAFR antagonists have also been shown to alter systemic immune effects (163). Additionally, PAFR antagonists have reduced experimental tumor repopulation both *in vitro* and *in vivo* (142, 164). These studies further illustrate the utility of PAFR antagonists as a pharmacological agent aimed at modulating the PAF/PAFR system and subsequent MVP release. Unfortunately, selective PAFR antagonists are not commercially available for clinical application for the targeting of MVP’s.

### Selective COX-2 Inhibitors

Nonsteroidal anti-inflammatory drugs (NSAIDs) are commercially accessible drugs utilized across the globe for anti-inflammatory, antipyretic, and analgesic use. Through inhibition of cyclooxygenase (COX) enzymes in the arachidonic acid metabolism pathway, these pharmaceutical agents decrease prostaglandin and thromboxane levels. Within this group of medications, COX-1 and COX-2 isoforms are inhibited to various degrees of selection by pharmaceutical agents. Of interest, our lab has shown the activation of epidermal PAFR leads to increased PAF, eicosanoids, COX-2, its product PGE2, arachidonic acid, IL-6, and IL-8. Furthermore, our lab’s findings with HaCaT and KB keratinocytes demonstrate that PAFR is sufficient to induce epidermal COX-2 production (165) Additionally, ultraviolet radiation, along with CPAF induced COX-2 activation, and subsequent PGE2 and IL-10 production, has been found to be inhibited by PAFR and COX-2 antagonists, further highlighting the linkage between PAF and its downstream effects with COX-2 (10, 17).

Due to the increased levels of COX-2 and prostaglandins in cutaneous malignancies, pharmacological intervention with COX-2 inhibitors for skin cancer has been examined in clinical trials and systematic reviews. In 2010, a double-blinded placebo-controlled trial on patients with pre-existing actinic keratoses found fewer incidences of squamous cell carcinomas and basal cell carcinomas in patients taking 200 mg of celecoxib twice daily (161). It has been hypothesized that these changes are linked in part to the impact of COX-2 inhibitors on UVB-based systemic immune suppression (42, 166). In particular with melanomas, COX-2 has been implicated as an important pro-immunosuppressive agent. COX-2 also enhances tumor-induced melanoma angiogenesis by increasing vascular endothelial growth factor (VEGF) through phosphoinositide 3-kinase (PI3K)/protein kinase C (PKC) mechanisms. Furthermore, there is emerging evidence correlating COX-2 with Breslow thickness and metastasis, indicating COX-2 roles in prognostic outcomes (136). One mechanism of interest is the role of COX-2 inhibitors on Treg cell depletion in PAFR pathways thus inhibiting tumor growth (42, 129). The woven nature of COX-2 and melanoma implicates roles for COX-2 inhibitors possibly impacting downstream effects of MVPs. Nonetheless, selective COX-2 inhibitors have associated cardiovascular risks which must be taken into account when considering a broad application in skin cancer (167, 168). In conclusion, targeted therapy of COX-2 offers a prospective mechanism to inhibit PAFR activation and MVP release.

## Conclusion

In conclusion, accumulating evidence has implicated the PAF family of mediators in UV cutaneous responses. Recent studies have suggested that their ability to generate and travel in MVP could provide a mechanism by which a highly potent yet metabolically labile family of lipids may leave the epidermis and thus impact the host. Moreover, it is likely that other agents can utilize subcellular particles such as MVP or exosomes to travel from the skin. These new areas of study have tremendous therapeutic implications, especially given the ability of aSMase inhibitors including FIASM agents such as imipramine and other FIASMs to potently block this pathway. The use of these pharmacologic tools can provide important insights into the roles of this pathway in UV as well as other environmental cutaneous insults.

## Author Contributions

TF, MG, GB, TW, and TN contributed equally to this work and wrote the first draft; JT and CR helped conceptualize the work and edited and provided further input. All authors contributed to the article and approved the submitted version.

## Funding

This research was supported in part by grants from the National Institutes of Health R01 HL062996 (JT), R01 ES031087 (JT and CR) and Veteran’s Administration Merit Award 5I01BX000853 (JT).

## Conflict of Interest

The authors declare that the research was conducted in the absence of any commercial or financial relationships that could be construed as a potential conflict of interest.

## Publisher’s Note

All claims expressed in this article are solely those of the authors and do not necessarily represent those of their affiliated organizations, or those of the publisher, the editors and the reviewers. Any product that may be evaluated in this article, or claim that may be made by its manufacturer, is not guaranteed or endorsed by the publisher.

## Abbreviations

UVB, Ultraviolet B; MVP, microvesicle particles; PAF, platelet-activating factor; PAFR, Platelet-activating factor receptor; GPC, glycerophosphocholine; PAF-AH, PAF acetylhydrolase; aSMase, acid sphingomyelinase.
